# Regrowth Patterns in Glioblastoma—Survival and Predictors

**DOI:** 10.1002/cam4.71764

**Published:** 2026-04-02

**Authors:** Jonas A. Feldheim, Jana Grieger, Teresa Schmidt, Christoph Oster, Julia J. Feldheim, Pia Wepner, Elke Hattingen, Carsten Hagemann, Ulrich Sure, Ulrich Herrlinger, Martin Stuschke, Björn Scheffler, Cornelius Deuschl, Christoph Kleinschnitz, Lazaros Lazaridis, Sied Kebir, Niklas Schäfer, Martin Glas

**Affiliations:** ^1^ Division of Clinical Neuro‐Oncology, Department of Neurology and Center for Translational Neuro‐ and Behavioral Sciences (C‐TNBS) University Medicine Essen, University Duisburg‐Essen Essen Germany; ^2^ Department of Neurology University Hospital Nuremberg, Paracelsus Medical University Nuremberg Germany; ^3^ German Cancer Consortium (DKTK) Partner Site University Medicine Essen Essen Germany; ^4^ DKFZ‐Division Translational Neurooncology at the West German Cancer Center (WTZ) DKTK Partner Site, University Medicine Essen, University Duisburg‐Essen Essen Germany; ^5^ Department of Neurosurgery University Hospital Essen Essen Germany; ^6^ Division of Clinical Neurooncology, Department of Neurology Medical Center Bonn Bonn Germany; ^7^ Institute of Neuroradiology Goethe University Hospital Frankfurt am Main Germany; ^8^ Section Experimental Neurosurgery, Department of Neurosurgery University Hospital Würzburg Würzburg Germany; ^9^ Department of Radiation Oncology University Hospital Essen Essen Germany; ^10^ Institute of Diagnostic and Interventional Radiology and Neuroradiology University Hospital Essen, University Duisburg‐Essen Essen Germany; ^11^ Department of Neurology University Hospital Knappschaftskrankenhaus Bochum, Ruhr University Bochum Bochum Germany

**Keywords:** distant, local, multicentric, multifocal, progression, recurrence, TTFields

## Abstract

Glioblastomas (GBM) can recur in different ways. While local recurrence is most common, some GBM recur at distant sites or simultaneously at multiple sites. However, the consequences of different regrowth patterns for the clinical course and the factors that might influence regrowth patterns or different modalities of recurrence remain unclear. We wondered (1) whether a more accurate analysis of regrowth patterns helps to detect subgroups of GBM patients, (2) if evaluation of relapse patterns correlates with differences in survival, and (4) whether we can identify predictors for distinct regrowth patterns. Therefore, we retrospectively collected demographic data, as well as tumor‐ and patient‐characteristics, from 251 patients treated at two institutions and characterized their recurrence patterns by analyzing Magnetic Resonance Imaging data. We observed distinct differences in patients' overall and progression‐free survival with respect to multicentric and multifocal recurrences, further supporting the hypothesis that these recurrences develop differently. Several tumor relapse patterns were associated with patients' progression‐free and overall survival (e.g., unifocal local and multicentric recurrences; *p* < 0.05), even when other known prognostic factors were taken into account. TTFields were associated with prolonged progression‐free survival (mPFS 7.2 months vs. 4.8 months, *p* = 0.03). They were predictive of a higher frequency of non‐local recurrence patterns (OR 0.16, *p* = 0.02) and longer time to development of a local recurrence after subtotal tumor resection (mPFS 11.1 months vs. 5.2 months; *p* = 0.01). This can be interpreted as a sign of improved local control.

## Graphical abstract

Multiple glioblastoma relapse patterns were associated with patients' progression‐free and overall survival, even when other known prognostic factors were taken into account. Treatment with Tumor Treating Fields was associated with prolonged progression‐free survival and predictive for a higher frequency of non‐local recurrence patterns after subtotal tumor resection, most likely due to improved local control.

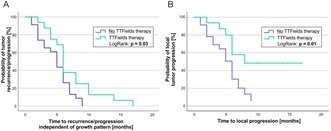

## 1 Introduction

Glioblastoma (GBM) is one of the most aggressive and common primary brain tumors of adults, making up around 50% of malignant brain tumors [1]. Despite an intense standard therapy, which encompasses surgical resection, radiation (RT) and chemotherapy (+/− Tumor Treating Fields (TTFields)), patients' prognosis remains poor [2]. Most patients are treated with temozolomide (TMZ)‐based chemotherapy, often referred to as “Stupp”‐scheme, named after the lead investigator of the underlying clinical trial published in 2005 [2]. However, newer treatment options, such as combined chemotherapy of TMZ and lomustine (CCNU), often named after the “CeTeG”‐trial, or the application of electric TTFields, have shown promising responses in suitable patients and, therefore, also found their way into clinical routine in recent years [3, 4]. However, despite recent advances, less than 5% of patients survive longer than 5 years after diagnosis [5]. Recurrence tends to be the rule rather than the exception, creating additional obstacles as relapses often gain in aggressiveness and further resistance to therapy [6]. GBM recurrence can occur in multiple ways [7]. Presumably, the main pattern of relapse is based on residual tumor cells near the edge of resection, rebuilding a malignant multicellular network close to the location of the primary tumor. Such growth pattern usually is classified as local or, in case of radiation, in‐field recurrence [8, 9, 10, 11, 12]. In contrast to most solid tumors, gliomas mainly migrate through perivascular spaces and the brain parenchyma and, therefore, do not depend on intravascular or lymphatic routes to spread [13]. Consequently, it is not uncommon to find single tumor cells in distant parts of the brain that may be able to initiate a remote or even multifocal progression [9]. Approximately 10% of relapses develop distant to the former tumor bed without any radiologically visible contact to the primary tumor localization. In contrast, 80%–90% of regrowth develops closely (< 2 cm) to the former resection cavity [12, 14, 15, 16, 17]. The longitudinal analyses of GBM and GBM subclones have become of particular interest in recent years. Though new mutations or changes of the methylome may occur, most recurrences appear to consist of multiple genetic subclones of the primary tumor and, thus, closely resemble the initial tumor [18, 19, 20, 21, 22]. So far, we cannot predict the emerging subclones, as they appear to develop stochastically due to early driver mutations [23]. However, interestingly, one subgroup of tumor recurrences stands out: Molecular analyses of GBM recurrences revealed that the fraction of initial tumor mutations preserved in the recurrent tumor was significantly lower in distant recurrences than in tumors with a local recurrence pattern [24]. Reasons for tumor regrowth are found in intrinsic characteristics of tumor biology and acquired resistance mechanisms leading to therapy failure. The definite distribution and nature of these cells are still unknown, but it has been proven that the growth pattern immensely affects therapeutic options and patients' outcomes [24, 25]. We believe that the detailed analysis of recurrence could give new insights into the distribution of these residual cells and might even help us to identify biological markers to predict tumor growth and adapt our therapy accordingly. Therefore, we asked (1) if a more accurate analysis of regrowth patterns helps to detect subgroups of GBM patients, (2) whether the evaluation of relapse patterns correlates with differences in survival, and (4) whether we can identify predictors for specific regrowth patterns. As an initial exploration with a collective of patients treated at the Division of Clinical Neurooncology, University of Essen yielded unexpected results, we aimed to verify the observations by analyzing an independent patient cohort treated at the Division of Clinical Neurooncology, University of Bonn. Rather than directly replicating our approach, we focused on patients treated in earlier years and were, therefore, able to challenge additional issues that could not sufficiently be answered with the initial cohort (e.g., regrowth pattern in partially resected tumor or multicentric regrowth).

## 2 Materials and Methods

### 2.1 Study Design

In this bicentric retrospective trial, we screened all patients that were treated in the Division for Clinical Neurooncology, Department of Neurology, University Medicine Essen, University Duisburg‐Essen, Essen, Germany, between January 1st, 2018 and December 31st, 2021 for the initial collective (Essen). Patients treated in the Division of Neurooncology, Department for Neurology, University Hospital Bonn, Bonn, Germany between January 1st, 2008 and December 31st, 2016 served as the verification cohort (Bonn). The Institutional Review Boards of the University of Bonn (written approval, no reference number allocated) and the University of Duisburg‐Essen (reference number: 21‐10248‐BO) approved the study. Patients were included in the trial if Magnet Resonance Imaging (MRI) scans were available at the time of first relapse. An additional early postoperative T1 gadolinium‐enhanced MRI within 72 h was preferable. We collected basic demographic and clinical data obtained within the framework of routine clinical assessment. Data collection included demographics, site of enhancing tumor (pre‐surgical and at the time of first relapse), pre‐ and postoperative Karnofsky performance status (KPS), the extent of resection, molecular parameters, date of first and second recurrence, first‐line and second‐line therapies and date of death or last seen alive.

### 2.2 Radiographic Assessment

Response assessment in neuro‐oncology (RANO) criteria were used to rule out pseudoprogression after radio‐chemotherapy [26, 27]. Radiographic examination was based on a modified nomenclature of Pope and colleagues [28]. As a first step, we established four different terms: “Local recurrence”, “distant recurrence”, “multicentric recurrence”, and “new contrast enhancing (CE) lesion in preexisting non‐CE‐lesion”. Patients developed “local recurrence” if new CE lesions occurred within a 3 cm margin of the former resection cavity. In the case of gross‐total resection, we called the pattern local recurrence (after gross‐total resection). In the case of subtotal resection, biopsy, or lesions at diagnosis that were not operated on, this pattern was described as local recurrence (progression of residual tumor). In contrast, “distant recurrence” implied remote CE tumor growth > 3 cm beyond the site at diagnosis. The term “multicentric recurrence” combines simultaneous local and distant recurrence patterns. Tumor growth was rated as “new CE‐lesion in preexisting non‐CE‐lesion” if a new CE lesion was observed in a prior hyperintense lesion on T2/FLAIR‐weighted imaging that had previously not been CE. If the MRI assessment displayed more than one of these patterns in a patient, we chose the most predominant pattern.

**FIGURE 1:**
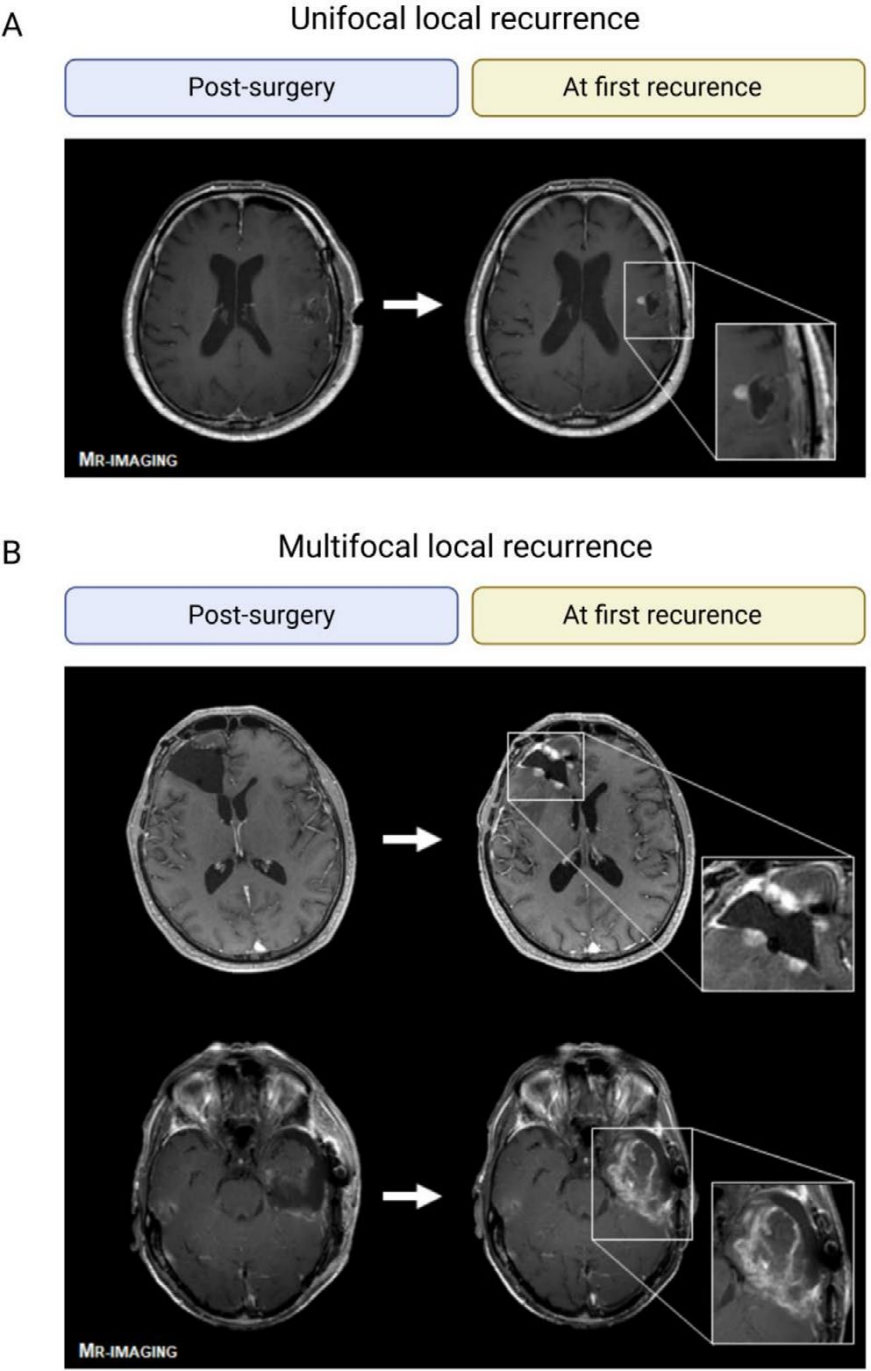
Recurrence patterns based on MR‐Imaging. Comparison of MR‐imaging post‐surgery and at first recurrence with examples of local unifocal recurrence with a single contrast‐enhanced lesion (A) or local multifocal recurrence with > 1 CE‐lesion (B, upper MRI) or even affection of the entire margins (B, lower MRI). Created in BioRender. Feldheim, J. (2023) BioRender.com/p08j063.

**FIGURE 2:**
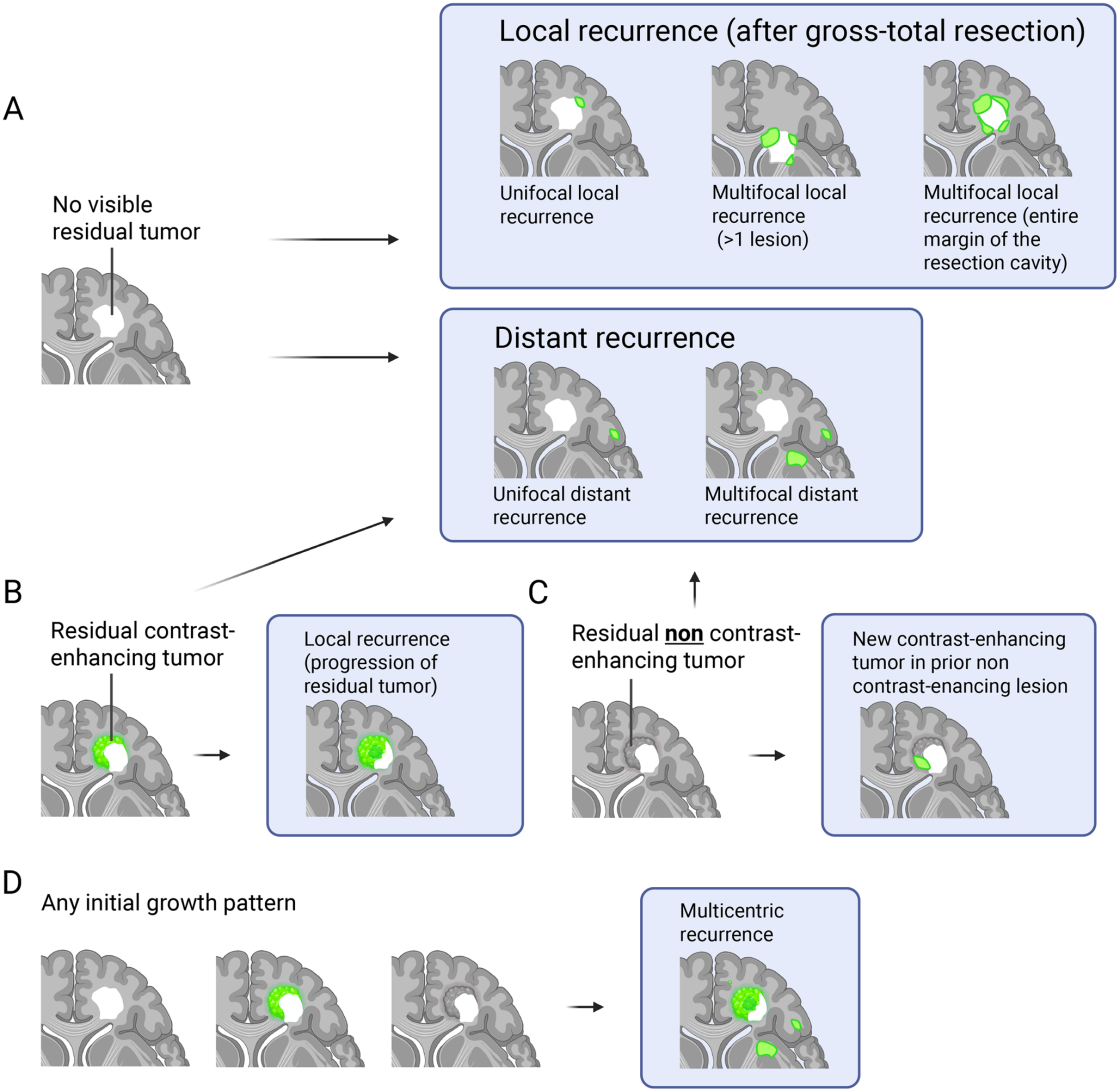
Schematic view of recurrence patterns. Scheme of different recurrence patterns after gross‐total resection (A), subtotal resection or stereotactic biopsy (B), either one with residual non‐contrast enhancing tumor (C) or any growth pattern with multicentric recurrence (D). Contrast‐enhancing tumor tissue is schematically shown in green. Created in BioRender.com. Feldheim, J. (2023) BioRender.com/e51x058.

In the second step, we subclassified the local and distant recurrences depending on the number of CE lesions and their distribution. In local recurrence (after gross‐total resection), we defined three categories: “unifocal local lesion”, “multifocal local (> 1 focal lesion)” and “multifocal local (lesions at the entire margin of the resection‐cavity)”. In the case of distant recurrence, we defined two categories, “unifocal distant lesion” and “multifocal distant lesions”. Figure 1 shows characteristic MRIs from patients with unifocal or multifocal local recurrence (after gross‐total resection). Figure 2 gives a schematic view of these patterns. This assessment was validated by an experienced neuroradiologist on a test set of 20 patients and continued by two independent raters. MRI controls were routinely performed every 12 weeks or earlier in case of clinical progression. In case of borderline or mixed cases, tumors were categorized according to their most dominant pattern, as classified by the raters, who reached a consensus in all cases. A distinction between progression/recurrence and pseudo‐progression was made based on the interdisciplinary tumor board's consensus to recommend either a follow‐up MRI (pseudo‐progression) or a change in treatment (progression or recurrence).

### 2.3 Statistical Analyses

We performed statistical analyses with IBM SPSS Statistics 28 (IBM Corporation, Armonk, NY, USA). We performed the Fisher exact test (Fisher) and Chi‐squared test (*χ* ^2^) to compare the group distribution. If alpha correction was required, it was performed according to Bonferroni. Kaplan–Meier survival estimates were calculated, and the log‐rank test (log‐rank) was used to compare the different GBM subgroups. Overall survival (OS) was calculated from the date of diagnosis (date of surgery) until death or for living patients until the date they were last seen alive. Progression‐free survival (PFS) was defined as the time from diagnosis (date of surgery) until the date of MRI‐confirmed progressive disease as verified by interdisciplinary consensus.

Regression analyses are based on the Cox proportional hazards model (Cox) for survival analyses and binary logistic regression (BLR) models for the prediction of specific recurrence patterns. In the survival models (Cox) we included the following variables (PFS and OS): age > 65 years, sex, O^6^‐Methylguanine‐DNA‐Methyltransferase (MGMT) promoter methylation, radiotherapy, TTFields, chemotherapy with the “Stupp” scheme, chemotherapy with the “CeTeG” scheme, KPS of at least 70%, local recurrence (after gross‐total resection), local recurrence (progression of residual tumor), distant recurrence, new CE‐lesion in preexisting (at the time of initial diagnosis) non‐CE‐lesion, unifocal local recurrence (after gross‐total resection), multifocal local recurrence (after gross‐total resection), multifocal local recurrence (lesions at the entire resection margin), unifocal distant recurrence, multicentric growth pattern as well as (only for OS) TTFields after first recurrence, resection after first recurrence, radiotherapy after first recurrence, TMZ re‐challenge after first recurrence, CCNU after first recurrence, bevacizumab after first recurrence.

We performed BLR with three different individual models for total resection, subtotal resection, and stereotactic biopsies. We selected local recurrence (after gross‐total resection) or local recurrence (progression of residual tumor) as the dependent variable, contrasting it with all other growth patterns. In the model, we included the variables: age > 65 years, sex, MGMT promoter methylation, radiotherapy, TTFields, chemotherapy with the “Stupp” scheme, chemotherapy with the “CeTeG” scheme, and KPS of at least 70%. For multivariate regression analyses (Cox and BLR), we estimated individual independent variables using an inclusion model before backward stepwise elimination (*p* = 0.05).

## 3 Results

### 3.1 Patients' Characteristics

The initial cohort (Essen) included 138 patients, the majority of whom were male. Patients were a median of 60 years old at the time of diagnosis and were in good clinical condition (median preoperative KPS 80%, median postoperative KPS 80%). Most patients received a macroscopic gross‐total resection (62.3%), radiotherapy (97.1%), and chemotherapy with TMZ only (66.7%). Additionally, 21.0% of patients were treated with a combination therapy of TMZ and CCNU following the CeTeG/NOA‐09 scheme [4]. One‐third of patients were treated with TTFields. Information on MGMT promoter methylation was obtained for all patients, and 39.9% were considered methylated. The cohort had a median PFS of 6 months and a median OS of 13 months.

The second cohort of patients (verification cohort from Bonn) consisted of 113 patients and included more males than females with a median age of 58. Patients were in a slightly more favorable clinical condition before and after primary surgery (median preoperative KPS 90%, median postoperative KPS 90%). Nearly half of the patients had a macroscopic gross‐total resection (45.0%), though subtotal resection was also common (43.1%), and patients mainly received standard therapy with radiotherapy and TMZ. MGMT promoter methylation status was available for all patients, and 32.7% harbored a methylated MGMT promoter region. For the whole cohort, the median PFS was 8 months, and the median OS was 15 months. Details are shown in the Supplement (Table S1). Most patients from the initial (Essen; 88.9%) and verification cohort (Bonn, 70.8%) developed local progression, divided almost equally between local recurrence (progression of residual tumor) and local recurrence (after gross‐total resection). Only two patients (1.4%) simultaneously developed distant and local lesions (multicentric) in the initial cohort (Essen) in contrast to 19 patients (16.8%) from the verification cohort (Bonn). Due to the small number, we refrained from a detailed subgroup analysis of multicentric growth in the initial cohort (Essen). A detailed compilation of growth patterns can be found in the Supporting Information (Table S2).

### 3.2 Multifocal Growth Was Common in Distant and Seldom in Local Recurrences

Next, we examined the growth pattern subgroups. Most local recurrences (after gross‐total resection) of the initial cohort (Essen) developed unifocal (*n* = 55; 84.6%) and only few developed multifocal (> 1 lesion or at the entire margin of the resection cavity; *n* = 5; 7.7%, each). In distant recurrences, unifocal growth was also the most common (*n* = 7, 63.6%). However, approximately one‐third of distant recurrences showed a multifocal pattern (*n* = 4, 36.3%). In the verification cohort (Bonn), non‐unifocal lesions were more common, allowing an additional comparison of local recurrence patterns between patients with total vs. subtotal resection. Out of 40 patients with local recurrence (after gross‐total resection), unifocal lesions appeared in 11 patients (27.5%), > 1 focal lesion was observed in 19 patients (47.5%), and the remaining 10 patients showed lesions at the entire margin of the resection cavity (25.0%). Patients with local recurrence (progression of residual tumor) after subtotal resection (*n* = 47) developed unifocal local lesions in 21.3% (*n* = 10), multifocal local lesions in 42.5% (> 1 lesion, *n* = 20) and 36.1% (lesions at the entire margin of the resection cavity, *n* = 17). Distant recurrence pattern was further analyzed in 27 patients (i.e., eight patients with distant regrowth and 19 with multicentric regrowth). Unifocal distant lesions occurred in 11 patients (40.7%), and > 1 focal distant lesion was observed in 16 patients (59.3%).

### 3.3 Recurrence Growth Pattern Was Associated With OS and PFS

Consecutively, we wondered whether the different growth patterns impacted patients' survival. A closer observation of our initial cohort (Essen) revealed that the patterns of recurrence, partly influenced by the extent of resection, were associated with both the PFS and the OS (*p* < 0.01, Table 1). Patients developed distant or local recurrence (after gross‐total resection) after a median time of 8 months. In contrast, local recurrence (progression of residual tumor) occurred after 5 months and CE‐lesions out of prior non‐CE tumor tissue even already after 3 months (Table 1). However, patients with a local recurrence (after gross‐total resection) lived significantly longer (median OS = 20 months, *p* < 0.01) than patients with a local recurrence (progression of residual tumor) (median OS = 10 months) and distant recurrence (median OS = 12 months, Table 1). Multicentric growth was excluded from this analysis due to a low total number (*n* = 2). Subgroups of patients with local or distant recurrences divided by unifocal or multifocal growth showed no significant difference in survival (Table 1).

**TABLE 1:** Correlation of relapse pattern and survival times of the initial cohort (Essen).

	*n*	%	Progression‐free survival			Overall survival		
			Median [months]	Quartiles [months]	Log‐rank	Median [months]	Quartiles [months]	Log‐rank
*Regrowth pattern* ^a^					*p* < 0.01			*p* < 0.01
Local recurrence (after gross‐total resection)	63	46.3	8	6–14		20	14–34	
Local recurrence (progression of residual tumor)	58	42.6	5	3–7		12	9–22	
Distant recurrence	9	6.6	8	5–9		10	9–16	
New CE lesion in prior non‐CE lesion	6	4.4	3	2–6		16	14–24	
*In case of local recurrence (after gross‐total resection)*					*p* = 0.53			*p* = 0.69
Unifocal lesion	55	84.6	8	5–14		20	14–34	
> 1 focal lesion	5	7.7	11	10–11		22	19–24	
Entire margin	5	7.7	7	6–7		25	20 – n.o.^b^	
*In case of distant recurrence*					*p* = 0.76			*p* = 0.88
Unifocal lesion	7	63.6	7	3–9		15	12–16	
> 1 focal lesion	4	36.3	5	5–9		24	14–26	

^a^ Two patients with simultaneous local and distant regrowth were excluded.

^b^ Not obtained as too many patients in the subgroup were censored due to still being alive at the end of the observation time.

In contrast, our verification cohort (Bonn) showed no significant differences in progression‐free survival with a tendency of longer PFS in distant recurrence (median 12 months) and CE‐lesions in prior non‐CE tumor tissue (median 15 months). Overall survival was significantly shorter in patients with multicentric regrowth (median OS = 8 months) compared to the other patterns (*p* < 0.01, Table 2). A closer observation revealed that patients with local unifocal growth had a significantly longer median PFS (14 months vs. 7 and 6 months, *p* < 0.05, log‐rank) and a tendency towards longer OS (49 months vs. 16 and 18 months, *p* = 0.07, log‐rank) compared to patients with a local recurrence (after gross‐total resection) and > 1 lesion or lesions at the entire margin (Table 2). In distant recurrences, we observed no significant differences regarding OS or PFS between patients with singular or multifocal recurrences (Table 2). As 47 patients of our verification cohort (Bonn) developed local recurrence (progression of residual tumor) after subtotal resection, we included them in a subgroup analysis, similar to the progression patterns subgroup analysis after local recurrence (after gross‐total resection). We observed that patients developed a unifocal progression after a significantly longer time than multifocal lesions or lesions at the entire resection margin (median PFS 13 months vs. 6 and 7 months, *p* < 0.05, log‐rank, Table 3). Progression patterns did not significantly affect survival, though there was a tendency towards longer OS in patients with unifocal local recurrence (progression of residual tumor) (median OS 21 months vs. 15 and 13 months, *p* > 0.05, log‐rank, Table 3).

**TABLE 2:** Correlation of relapse pattern and survival times of the verification cohort (Bonn).

	*n*	%	Progression‐free survival			Overall survival		
			Median [months]	Quartiles [months]	Log‐rank	Median [months]	Quartiles [months]	Log‐rank
*Regrowth pattern*					*p* = 0.39			*p* = 0.06
Local recurrence (after gross‐total resection)	31	27.4	8	5–11		18	14–38	
Local recurrence (progression of residual tumor)	49	43.4	7	6–10		18	13–32	
Distant progression	7	6.2	12	6–17		21	11 – n.o.^b^	
New CE lesion in prior non‐CE lesion	7	6.2	15	8–20		20	16–28	
Multicentric^a^	19	16.8	8	6–13		12	10–21	
*In case of local recurrence (after gross‐total resection)*					*p* < 0.05			*p* = 0.07
Unifocal lesion	7	22.6	14	8–42		49	20 – n.o.^b^	
> 1 focal lesion	16	51.6	7	3–9		16	12–20	
Entire margin	8	25.8	6	5–7		18	14–18	
*In case of distant recurrence*					*p* = 0.91			*p* = 0.38
Unifocal lesion	4	57.1	6	5–12		11	10–21	
> 1 focal lesion	3	42.9	12	8–17		n.o. ^2^	12 – n.o.^b^	

^a^ Simultaneous local and distant regrowth as an independent group excluded from other subgroups.

^b^ Not obtained (n.o.) as too many patients in the subset were censored due to still being alive at the end of the observation time.

**TABLE 3:** Correlation of relapse pattern and survival times of patients of the verification cohort with subtotal resection that developed a local recurrence (progression of residual tumor) (Bonn).

	*n*	%	Progression‐free survival			Overall survival		
			Median [months]	Quartiles [months]	Log‐rank	Median [months]	Quartiles [months]	Log‐rank
*In case of local recurrence (progression of residual tumor) after subtotal resection* ^a^					*p* < 0.05			*p* = 0.18
Unifocal lesion	10	21.3	13	10–18		21	18 – n.o.^b^	
> 1 focal lesion	20	42.6	6	4–9		15	9–32	
Entire margin	17	36.1	7	6–9		13	12–22	

^a^ Simultaneous local and distant regrowth as an independent group excluded from other subgroups.

^b^ Not obtained (n.o.) as too many patients in the subset were censored due to still being alive at the end of the observation time.

To determine whether the GBM relapse growth patterns were independent predictors for OS and PFS we performed stepwise backward elimination multivariate Cox regression models. In the model of the initial cohort (Essen), we verified multiple recurrence growth patterns to be independent predictors for PFS and OS. Other independent predictors were the MGMT promoter methylation and chemotherapy (Stupp and CeTeG) for PFS (Table S3), and the MGMT promoter methylation, radiotherapy after diagnosis and therapy after recurrence (surgery, radiotherapy) for OS (Table S4). In multivariate analyses of the verification cohort (Bonn), only MGMT promoter methylation remained an independent predictor for PFS (Table S5). MGMT promoter methylation, patients' sex, recurrence pattern (unifocal local, multicentric) and therapy after recurrence (surgery) were independently associated with OS (Table S6).

### 3.4 Treatment With TTFields Was Associated With Less Local Recurrence and Longer PFS After Subtotal Resection

**FIGURE 3:**
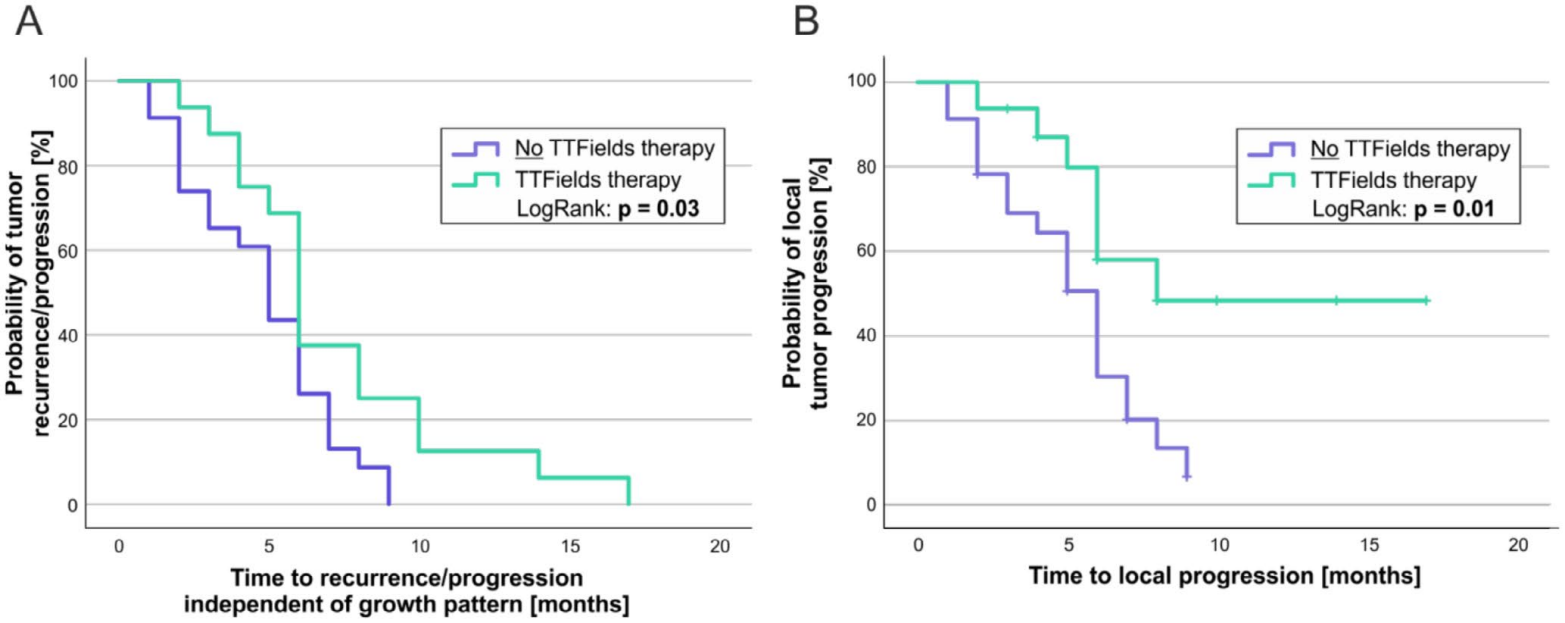
Kaplan–Meier analyses of PFS of patients after subtotal resection depending on whether they were (green, *n* = 16) or were not (blue, *n* = 23) treated with TTFields: PFS independent of patients' recurrence pattern (event = progression/recurrence) (A). PFS until local recurrence (progression of residual tumor) (event = local recurrence (progression of residual tumor); patients with other growth patterns were censored when the different types of recurrence occurred) (B).

To examine possible associations of clinical and therapeutic characteristics, we divided our patient cohorts by the extent of resection after surgery. In our initial cohort (Essen), none of the clinical parameters predicted local recurrence in patients with a gross‐total resection or stereotactic biopsy. However, in the subcohort with subtotal resection, treatment with TTFields significantly predicted the likelihood of a local recurrence (progression of residual tumor) as compared to distant recurrences and progression of a CE tumor in prior non‐CE tissue (Odds ratio: 0.16, 95% CI 0.04–0.70; *p* = 0.02; Hosmer‐Lemeshow test: *p* = 1.0, Cox & Snell *R* ^2^ = 0.15, Nagelkerke *R* ^2^ = 0.21). Only seven out of sixteen patients (43.8%) who were treated with TTFields developed local progress, whereas 19 out of 23 (82.6%) who were not treated with TTFields did (*χ* ^2^, *p* = 0.01; Fisher, *p* = 0.02). In our verification cohort (Bonn), only one patient was treated with TTFields. It deserves to be noted that this patient did not develop a local recurrence (progression of residual tumor), but a CE recurrence at residual non‐CE tumor tissue after a stereotactic biopsy. Apart from that, all three models of the verification cohort (Bonn) did not reveal a significant predictor. Interestingly, TTFields treatment was associated with a longer PFS in the subgroup of patients that received subtotal tumor resection (mean PFS of 7.2 vs. 4.8 months, LogRank *p* = 0.03, Figure 3A). This effect appeared to be especially prominent in the PFS until local recurrence (progression of residual tumor) (mean PFS 11.1 vs. 5.2 months, LogRank *p* = 0.01, Figure 3B) and was associated with a slight tendency towards longer OS (mean PFS 16.8 vs. 13.8 months, LogRank *p* > 0.05).

In the subgroup of patients that developed a local recurrence (after gross‐total resection), we observed MGMT promoter methylation as an independent predictor (Odds ratio: 9.2, 95% CI 1.8–46.8, *p* < 0.01; Cox & Snell *R* ^2^ = 0.20; Nagelkerke *R* ^2^ = 0.28) for a unifocal local recurrence (after gross‐total resection) as compared to multifocal local growth and growth at the entire resection margin. Seven out of eleven (63.6%) patients with MGMT hypermethylation developed a unifocal recurrence, whereas only 4/29 (13.8%) without MGMT did (*χ* ^2^, *p* < 0.01; Fisher *p* < 0.01).

Similarly, the sub‐collective of patients that developed a local recurrence (progression of residual tumor) after subtotal resection revealed MGMT promoter methylation and chemotherapy according to “Stupp” as independent predictors for unifocal local growth (Odds ratio of MGMT promoter methylation, 14.0, 95% CI 1.4–143.0, *p* = 0.03; Odds ratio of chemotherapy according to Stupp, 0.05, 95% CI 0.01–0.57 *p* = 0.02; Cox & Snell *R* ^2^ = 0.26; Nagelkerkes *R* ^2^ = 0.39). Five out of 13 (38.5%) patients with MGMT promoter methylation developed unifocal local recurrence (progression of residual tumor) compared to 5/34 (14.7%) without MGMT promoter methylation (*χ* ^2^, *p* = 0.08, Fisher *p* = 0.11). The same was true for 6/40 (15.0%) patients treated with TMZ chemotherapy, according to Stupp, whereas 4/7 (57.1%) patients with other systemic treatment developed a unifocal local recurrence (progression of residual tumor) (*χ* ^2^ 0.01, Fisher 0.03). In the sub‐collective of our initial cohort (Essen) with local recurrence (after gross‐total resection), we observed a similar (statistically non‐significant) tendency, as 23/24 (95.8%) patients with MGMT promoter hypermethylation developed a unifocal recurrence, while 26/32 (81.2%) of those without hypermethylation did (*χ* ^2^, *p* > 0.05; Fisher *p* > 0.05).

## 4 Discussion

### 4.1 Key Findings

Our analyses of GBM regrowth patterns verified distinct differences in development and survival between specific relapse patterns (e.g., multifocal and multicentric growth, local and distant recurrence). In contrast, others appeared to be different stages of a similar development (e.g., multifocal local recurrences > 1 lesion and multifocal local recurrences with lesions at the entire margin of the resection cavity). Multiple growth patterns were associated with shorter or longer PFS or OS, even when other prognostic factors like the MGMT promoter methylation or applied therapy were considered. However, the only significant independent predictor of recurrence patterns in our study was TTFields therapy, which appeared to suppress local recurrences. Our patient cohorts' demographic data and patient characteristics were representative of the GBM population (Supporting Information S1 and Table S1).

### 4.2 Multicentric and Multifocal Recurrence Differ in PFS and OS, Supporting Their Hypothesized Different Origin

Multifocal/multicentric growth at GBM diagnosis has been made out as an independent prognostic factor associated with shorter OS [25, 29, 30, 31]. Most studies distinguish between multicentric GBM, hypothesized to constitute simultaneously but independently developing lesions from a monoclonal source, and multifocal GBM, believed to consist of foci that appear independent on MRI but are interconnected [8, 32, 33, 34, 35]. However, distinguishing criteria or diagnostic standards for classification have not been commonly defined, and most observations are based on primary multicentric/multifocal rather than recurrence growth patterns investigated in our trial [36]. Interestingly, Syed et al. reported that multicentric/multifocal growth in recurrence appeared to be independent of the primary growth pattern, highlighting that the mechanisms and effects of multicentric/multifocal growth are poorly understood and an interesting starting point for future investigations [37].

Though the number of multicentric recurrences in our initial cohort (Essen) was too low (*n* = 2) to draw reliable conclusions and were not analyzed as an independent group, multicentric recurrences were associated with a similar PFS but significantly worse OS compared to the other growth patterns in the verification cohort (Bonn), thereby verifying the observations previously published by Syed et al. [37].

Interestingly, this stood in contrast to our observations on multifocal local recurrence (after gross‐total resection). After gross‐total, as well as subtotal resection, the patients from our verification cohort (Bonn) both developed their multifocal (> 1 lesion/entire margin) local recurrences (after gross‐total resection) significantly faster than unifocal recurrences with a tendency towards shorter OS. In our initial cohort (Essen), the multifocal subgroups only consisted of five patients, considerably limiting their informative value. As we observed similar OS and PFS between multifocal local recurrences (after gross‐total resection) with > 1 focal lesion and lesions of the entire margin of the resection cavity, we hypothesize that distinguishing between the number of multifocal lesions in local recurrence (after gross‐total resection) might not be necessary. The subgroups of distant recurrences based on unifocal/multifocal growth were low in both cohorts. Therefore, we cannot assess if the similar OS and PFS between both groups was due to a similar clinical course or a low sample number.

### 4.3 Development of Distant Recurrences Prolonged Survival and Might (Partly) Be Explained by Local Tumor Control

How the prognosis of distant recurrences compares to local recurrences (after gross‐total resection) is still unclear and difficult to assess, as there is no unequivocal definition of distant recurrences. A common point of most definitions, however, is that distant recurrences are believed to appear out of the field of radiation and the resection cavity. The occurrence of distant recurrence could be explained by more aggressive tumor growth or better local tumor control, leading to prolonged survival and allowing distant recurrences to develop. Exemplary for the second hypothesis, Mendoza et al. showed that patients with a distant recurrence lived significantly longer in median (21.4 months) than patients of their cohort with an “in‐field” recurrence (12.1 months) after stereotactic radiosurgery [38]. Guberina et al. [39] describe a similar OS between patients with an in‐field and out‐field recurrence, yet also state an association with out‐field recurrences and multifocal/multicentric growth, as well as a longer PFS for out‐field recurrences. We observed similar associations in our verification cohort (Bonn).

In contrast, our initial cohort (Essen), which slightly overlaps with the cohort of Guberina et al., only mirrored this observation between local recurrences (progression of residual tumor) and distant recurrences. Local recurrences (after gross‐total resection) had a similar PFS but significantly longer OS than distant recurrences [39]. As local recurrences (after gross‐total resection) did, by definition, develop after gross‐total resection, and patients from our initial cohort (Essen) were treated only in the last few years (beginning in 2018, compared to 2008 [Bonn] and 2007 [Guberina et al.]), we attribute this difference to the advances made in neurosurgery [39]. Multivariate survival analyses verified that tumor regrowth patterns were significant independent predictors for PFS and OS, alongside known prognostic factors, such as the MGMT promoter methylation and therapy after diagnosis and recurrence [37, 40, 41]. The multivariate model of the verification cohort (Bonn) revealed patients' sex as an independent predictor for survival, which is coherent with previous reports of a female survival advantage that might be caused by a sex‐specific immune response [42, 43, 44]. However, it is important to note that while multivariable analyses help reduce bias from potential confounders, they cannot eliminate it. Different baseline characteristics not evaluated in this retrospective design (such as molecular features, tumor location, or treatment toleration) might have influenced our results.

### 4.4 TTFields Were Associated With Longer PFS, Especially Regarding Local Recurrence After Subtotal Resection

A significant focus of our project also lay on potential predictors for different growth patterns. Therefore, we divided our cohorts by the extent of resection. We observed that MGMT promoter methylation was associated with tumor recurrence patterns, as previously described [17, 45, 46]. However, in contrast to previous observations, MGMT promoter methylation was not a predictor for distant progression but for unifocal local recurrence (progression of residual tumor) [17, 45, 46]. While appearing contradictory at first sight, the higher frequency of distant recurrences in MGMT hypermethylated tumors has been attributed to better local control and prolonged survival [17, 45, 46]. While previous observations were mainly divided between local and distant recurrences, we hypothesize that our subclassification led to unifocal local recurrences (after gross‐total resection) being associated with MGMT promoter methylation, as this was the subcategory with superior local control and longer survival.

Interestingly, treatment with TTFields emerged as an independent predictor in the subcohort after subtotal resection. As it was associated with a longer PFS in general, and especially with a longer PFS until local recurrence (progression of residual tumor), we hypothesize that patients in this subgroup treated with TTFields may have displayed superior local tumor control. Consecutively, we observed more distant recurrences and CE lesions out of non‐CE tumor tissue after TTFields whose PFS did not significantly differ between both groups. These observations agree with the post hoc analyses of the EF‐14 cohort, where a significantly higher frequency of distant recurrences with a longer PFS in the TTFields arm was demonstrated [17]. Our observations support the conclusions of Glas et al. that TTFields might suppress local tumor growth and lead to other types of recurrence due to an extension of patient survival [17]. While Glas et al. only distinguished between distant and local recurrence (after gross‐total resection), we also observed that the other regrowth patterns in our classification, such as CE lesions developing from prior non‐CE tumor tissue (not investigated in Glas et al.), were more common after TTFields therapy. Furthermore, multiple patients in our study who were treated with TTFields after gross‐total resection belonged to the EF‐14 trial cohort, on which Glas et al. based their conclusions. As we observed a weak tendency in this subset but a strong effect in patients after subtotal resection, we hypothesize that the local tumor control of TTFields might be even greater when there is a contrast‐enhancing residual tumor that can be planned as the target area. Still, our observations are based on a subgroup that this study was not designed to investigate, and follow‐up projects will be required to verify our observations.

The following limitations must be considered: (1) Our data are based on two centers only and were retrospectively acquired. (2) A selection bias cannot be excluded, and important confounders might not have been evaluated due to the retrospective and observational study design. (3) Both cohorts were deliberately recruited in different timeframes. Still, they showed that regrowth patterns might be primarily influenced by therapeutic and diagnostic standards, thereby limiting the extent to which our observations can be generalized. (4) Standards for the definition of recurrence growth patterns vary, creating an inhomogeneity between different trials. (5) Despite being evaluated by RANO criteria, pseudoprogression cannot be ruled out with absolute certainty and might have biased our findings. Additionally, radiation‐related changes and overlapping recurrence patterns might have been misclassified due to their inherently difficult interpretation, potentially influencing the survival comparisons. (6) As shown and discussed above, tumor growth patterns may be influenced by therapy, which includes minor differences with potentially large consequences, such as the cycles of chemotherapy, extent of the radiation field, and extent of the surgical resection. Therefore, we cannot conclusively determine whether the observed changes are attributable to cell biology or to secondary factors. Also, MRI follow‐up standards may have influenced the results.

## 5 Conclusions

Multicentric and multifocal recurrences indicated distinct differences in OS and PFS, further supporting the hypothesis that they develop differently. Tumor relapse patterns were associated with patients' progression‐free and overall survival, even when other known prognostic factors were taken into account. However, unknown confounders, the observational study design, and classification uncertainties might have influenced our results. TTFields were associated with prolonged progression‐free survival and predictive of a higher frequency of non‐local recurrence patterns after subtotal tumor resection. This can be hypothesized to be a sign of improved local control.

## Author Contributions

**Jonas A. Feldheim:** data curation (lead), formal analysis (lead), investigation (lead), methodology (equal), software (equal), writing – original draft (lead), writing – review and editing (equal). **Jana Grieger:** data curation (lead), formal analysis (equal), investigation (lead), methodology (supporting), software (equal), writing – original draft (equal), writing – review and editing (supporting). **Teresa Schmidt:** formal analysis (equal), investigation (supporting), methodology (supporting), software (supporting), validation (equal), visualization (equal), writing – review and editing (supporting). **Christoph Oster:** conceptualization (supporting), formal analysis (supporting), investigation (equal), methodology (supporting), validation (supporting), visualization (supporting), writing – review and editing (equal). **Julia J. Feldheim:** conceptualization (supporting), formal analysis (equal), investigation (equal), methodology (supporting), software (supporting), visualization (supporting), writing – review and editing (supporting). **Pia Wepner:** investigation (lead), methodology (equal), visualization (lead). **Elke Hattingen:** formal analysis (supporting), project administration (supporting), resources (supporting), supervision (supporting), validation (lead), writing – review and editing (supporting). **Carsten Hagemann:** conceptualization (supporting), funding acquisition (supporting), methodology (supporting), validation (supporting), writing – review and editing (equal). **Ulrich Sure:** conceptualization (equal), project administration (supporting), resources (equal), validation (equal), writing – review and editing (supporting). **Ulrich Herrlinger:** conceptualization (lead), funding acquisition (equal), project administration (lead), resources (equal), supervision (equal), validation (equal), writing – review and editing (equal). **Martin Stuschke:** conceptualization (supporting), funding acquisition (supporting), project administration (supporting), validation (supporting). **Björn Scheffler:** conceptualization (supporting), investigation (supporting), validation (supporting), visualization (supporting), writing – review and editing (supporting). **Cornelius Deuschl:** funding acquisition (supporting), methodology (equal), project administration (supporting), software (supporting), validation (lead). **Christoph Kleinschnitz:** formal analysis (supporting), project administration (supporting), resources (equal), supervision (equal), validation (equal). **Lazaros Lazaridis:** formal analysis (supporting), investigation (supporting), methodology (supporting), software (supporting). **Sied Kebir:** formal analysis (equal), funding acquisition (supporting), resources (equal), supervision (equal), visualization (equal), writing – review and editing (supporting). **Niklas Schäfer:** conceptualization (lead), funding acquisition (lead), investigation (equal), methodology (equal), project administration (equal), resources (equal), supervision (lead), validation (equal), writing – original draft (equal), writing – review and editing (equal). **Martin Glas:** conceptualization (equal), formal analysis (supporting), funding acquisition (equal), methodology (equal), project administration (lead), resources (lead), supervision (lead), validation (lead), writing – original draft (supporting), writing – review and editing (lead).

## Funding

This work was supported by the UMEA Junior Clinician Scientist Grant of the Medical Faculty, University Duisburg‐Essen for this project. Otherwise, no external funding was received.

## Ethics Statement

The study was conducted in accordance with the Declaration of Helsinki, and was approved by the Institutional Review Board of the University of Bonn (written consent, no reference number allocated) and the University of Duisburg‐Essen (reference number: 21‐10248‐BO).

## Conflicts of Interest

Christoph Oster has received travel support from Novocure, honoria by Horizon and Novocure and a research grant by Servier. Carsten Hagemann has received a research grant, travel grants, and speakers' honoraria from Novocure. Ulrich Herrlinger reports consulting fees from Medac, OncomagnetX, Servier, Bayer and lecture honoraria from Medac and Bayer. Lazaros Lazaridis received honoraria and travel support from Novocure. Niklas Schäfer received honoraria from Servier. Martin Glas has received research grants from Novocure. He has received honoraria from Roche, Seagan, Servier, Novartis, UCB, Abbvie, Daiichi Sankyo, Bayer, Janssen‐Cilag, Kyowa Kirin, Medac, Merck, and Novocure. He has received travel support from Novocure and Medac.

## Supporting information


**Table S1:** Clinical parameters of GBM patients from the initial and verification cohort. ^1^Not determined/known for 1 patient; ^2^Not obtained in 17 patients; ^3^Not obtained in 15 patients; ^4^Missing information for 27 patients; ^5^Missing information for 8 patients; KPS, Karnofsky Performance Status Scale; n.o., not obtained; MGMT, O^6^‐methylguanine‐DNA‐methyltransferase; TTFields, Tumor Treating Fields; Stupp, Chemotherapy following the Stupp procedure; CeTeG, Chemotherapy following the CeTeG procedure; TMZ, Temozolomide; PFS, Progression‐free survival; CCNU, Lomustine; OS, overall survival.
**Table S2:** Relapse growth patterns of GBM patients from the initial and verification cohort. ^1^Two patients with simultaneous local and distant regrowth were excluded from the initial cohort (Essen). ^2^Simultaneous local and distant regrowth as an independent group excluded from other subgroups.
**Table S3:** Multivariate analysis of independent predictors/associations with PFS of our initial cohort (Essen). Cox regression model, stepwise backward elimination. CI, confidence interval; HR, hazard ratio; PFS, progression‐free survival.
**Table S4:** Multivariate analysis of independent predictors for OS of our initial cohort (Essen). Cox regression model, stepwise backward elimination. Variables included are stated in the Materials and Methods section. ^1^Age (> 65 years) was eliminated in the last step of the model (*p* = 0.052). CI, confidence interval; HR, hazard ratio; OS, overall survival; progression‐free survival.
**Table S5:** Multivariate analysis of independent predictors/associations with PFS of our verification cohort (Bonn). Cox regression model, stepwise backward elimination. Variables included are stated in the Materials and Methods section. 1Unifocal local recurrence (after gross‐total resection) (yes) was eliminated in the last step of the model (p = 0.062). CI, confidence interval; HR, hazard ratio; PFS, progression‐free survival.
**Table S6:** Multivariate analysis of independent predictors for OS of our verification cohort (Bonn). Cox regression model, stepwise backward elimination. Variables included are stated in the Materials and Methods section. CI, confidence interval; HR, hazard ratio; OS, overall survival; progression‐free survival.

## Data Availability

The data that support the findings of this study are available on request from the corresponding author. The data are not publicly available due to privacy and ethical restrictions.
