# Supplementary material for: Regrowth Patterns in Glioblastoma—Survival and Predictors
Source: Cancer Med. 2026 Apr 2;15(4):e71764. doi: 10.1002/cam4.71764 (PMC13052026; doi:10.1002/cam4.71764)
Supplement: Supplementary file 1 — Table S1: Clinical parameters of GBM patients from the initial and verification cohort. 1Not determined/known for 1 patient; 2Not obtained in 17 patients; 3Not obtained in 15 patients; 4Missing information for 27 patients; 5Missing information for 8 patients; KPS, Karnofsky Performance Status Scale; n.o., not obtained; MGMT, O6‐methylguanine‐DNA‐methyltransferase; TTFields, Tumor Treating Fields; Stupp, Chemotherapy following the Stupp procedure; CeTeG, Chemotherapy following the CeTeG procedure; TMZ, Temozolomide; PFS, Progression‐free survival; CCNU, Lomustine; OS, overall survival. Table S2: Relapse growth patterns of GBM patients from the initial and verification cohort. 1Two patients with simultaneous local and distant regrowth were excluded from the initial cohort (Essen). 2Simultaneous local and distant regrowth as an independent group excluded from other subgroups. Table S3: Multivariate analysis of independent predictors/associations with PFS of our initial cohort (Essen). Cox regression model, stepwise backward elimination. CI, confidence interval; HR, hazard ratio; PFS, progression‐free survival. Table S4: Multivariate analysis of independent predictors for OS of our initial cohort (Essen). Cox regression model, stepwise backward elimination. Variables included are stated in the Materials and Methods section. 1Age (> 65 years) was eliminated in the last step of the model (p = 0.052). CI, confidence interval; HR, hazard ratio; OS, overall survival; progression‐free survival. Table S5: Multivariate analysis of independent predictors/associations with PFS of our verification cohort (Bonn). Cox regression model, stepwise backward elimination. Variables included are stated in the Materials and Methods section. 1Unifocal local recurrence (after gross‐total resection) (yes) was eliminated in the last step of the model (p = 0.062). CI, confidence interval; HR, hazard ratio; PFS, progression‐free survival. Table S6: Multivariate analysis of independent predictors [file CAM4-15-e71764-s001.docx]

Supplementary Material

**Regrowth pattern in glioblastoma – Survival and predictors**

S1. *Demographic data and characteristics of patient cohorts were representative of the GBM population*

Consistent with the statistical report of the Central Brain Tumor Registry of the United States (CBTRUS), the slight majority of patients from both cohorts were male. However, the patients' median age (60 and 58 years) was slightly younger than 65 years ^47^. Macroscopic gross-total resection was more often achieved in our initial cohort (Essen), due to advances in operation techniques, since the verification cohort (Bonn) included patients going back to 2008. Similarly, new therapeutic options, such as TTFields or the CeTeG scheme for chemotherapy, were more common in the initial cohort (Essen) with patients treated more recently. As reported in previously published patient cohorts, 30-40% of patients had MGMT promoter hypermethylation ^21, 48, 49^. The median OS of 13 and 15 months is above the "real-life" OS as reported in the CBTRUS report (8 months, GBM independent of MGMT promoter methylation) and study cohort from the EORTC trial 26981/22981 ^48, 50^ (12.7 months, RT+TMZ; 11.8 months, RT), yet slightly lower than the study population of the EF-14 trial (16.9 months, RT+TMZ+TTFields; 14.7 months, RT+TMZ) ^3^. Both Departments participating in this study are large national centers with multiple patients included in clinical trials and, presumably, a high standard of care. Therefore, the OS is within the expected variance. We conclude that both patient cohorts represent the general population of GBM patients.

In our initial cohort, local recurrence (after gross-total resection) (mostly unifocal) and local recurrence (progression of residual tumor) were common and other growth patterns only appeared rarely. In contrast, a significant proportion of our verification cohort (Bonn) patients showed regrowth at multiple localizations. To put these differences into perspective, it is essential to note that both patient cohorts were recruited in different timeframes, and the observed differences are most likely a result of therapeutic and diagnostic advances, e.g., earlier detection of tumor regrowth and better detection of communication between lesions ^32^. For instance, gross-total resection, CeTeG chemotherapy, and treatment with TTFields, which have all been proven to aid in tumor control, were more common in the initial cohort (Essen). Similarly, one might argue that the shorter PFS of the initial cohort (Essen) might be due to a *lead time bias by an* earlier diagnosis of recurrence, which would also explain why multifocal (and multicentric) growth was rarely observed in our initial cohort (Essen).

| **Patients' Characteristics** | **Initial cohort (Essen; n=138)** | |  | **Verification cohort (Bonn; n=113)** |
| --- | --- | --- | --- | --- |
| **Sex (n, %)** | Female: 68 / 49.3%  Male: 70 / 50.7% |  | | Female: 38 / 33.6%  Male: 75 / 66.4% |
| **Age (median, quartiles)** | 60 years (53 – 69 years) |  | | 58 years (51 - 67 years) |
| **Preoperative KPS (median, quartiles)** | 80% (80 - 90%)^1^ |  | | 90% (80 – 90%)^2^ |
| **Postoperative KPS (median, quartiles)** | 80% (80 - 90%)^1^ |  | | 90% (70 – 90%)^3^ |
| **Localization at diagnosis (n, %)** | Frontal: 30 / 21.7%^1^  Occipital: 5 / 3.6%  Temporal: 38 / 27.5%  Parietal: 15 / 10.9%  Multicentric: 44 / 31.9%  Others: 5 / 3.6% |  | | Local: 100 / 88.5%^1^  Multicentric: 12 / 10.6% |
| **MGMT promoter methylation (n, %)** | Unmethylated: 83 / 60.1%  Methylated: 55 / 39.9% |  | | Unmethylated: 76 / 67.3%  Methylated: 37 / 32.7% |

*Table continued on next page*

| **Therapy & Survival** | **Initial cohort (Essen; n=138)** | **Verification cohort (Bonn; n=113)** |
| --- | --- | --- |
| **Primary treatment  (independent of TTFields)** | Stupp: 92 / 66.7%  CeTeG: 29 / 21.0%  Other chemotherapy: 4 / 2.9% No chemotherapy: 13 / 9.4% | Stupp: 93 / 82.3%  CeTeG: 4 / 3.5%  Other chemotherapy: 8 / 7.1% No chemotherapy: 8 / 7.1% |
| **Cycles of TMZ before relapse  (median, quartiles)** | 2 cycles (0 – 4.5 cycles) | 6 cycles (3 – 6 cycles)^4^ |
| **Extent of resection (n, %)** | Biopsy: 13 / 9.4%  Subtotal resection: 39 / 28.3%  Gross-total resection: 86 / 62.3% | Biopsy: 12 / 10.6%  Subtotal resection: 55 / 48.7%  Gross-total resection: 46 / 40.1% |
| **Radiotherapy (n, %)** | No radiotherapy: 4 / 2.9% Hypofractionated: 22 / 15.9%  Conventional: 112 / 81.2% | No radiotherapy: 0 / 0.0%  Radiotherapy: 113 / 100.0% |
| **Treatment with TTFields** | No TTFields therapy: 92 / 66.7%  TTFields therapy: 46 / 33.3% | No TTFields therapy: 112 / 99.1%  TTFields therapy: 1 / 0.9% |
| **PFS (median, quartiles)** | 6 months (4 - 10 months) | 8 months (6 - 13 months) |
| **Surgical intervention after relapse (n, %)** | No surgery: 81 / 58.7%  Resection: 57 / 41.3% | No surgery: 86 / 81.9%^5^  Resection: 19 / 18.1% |
| **Chemotherapy after relapse (n, %)** | Best supportive care: 13 / 9.4%  No chemotherapy: 11 / 8.0%  TMZ: 27 / 19.6%  CCNU: 59 / 42.8%  Bevacizumab: 15 / 10.9%  Regorafenib: 1 / 0.7%  Other systemic therapy: 12 / 8.7% | Best supportive care: 9 / 8.6%^5^  No chemotherapy: 7 / 6.7%  TMZ: 18 / 17.1%  CCNU: 31 / 29.5%  Bevacizumab: 17 / 16.2%  Regorafenib: 0 / 0.0%  Other systemic therapy: 23 / 21.9% |
| **Radiotherapy after relapse (n, %)** | No radiotherapy: 111 / 80.4 %  Radiotherapy: 27 / 19.6 % | No radiotherapy: 90 / 85.7 %^5^  Radiotherapy: 15 / 14.3 % |
| **TTFields after relapse (n, %)** | No TTFields therapy: 102 / 73.9%  TTFields therapy: 36 / 26.1% | No TTFields therapy: 105 / 100.0%^5^ TTFields therapy: 0 / 0.0% |
| **OS (median, quartiles)** | 13 months (9 - 19 months) | 15 months (11 - 21 months) |
|  |  |  |

**Table S1.** Clinical parameters of GBM patients from the initial and verification cohort. ^1^ Not determined/known for 1 patient; ^2^ Not obtained in 17 patients; ^3^ Not obtained in 15 patients; ^4^ Missing information for 27 patients; ^5^ Missing information for 8 patients; KPS, Karnofsky Performance Status Scale; n.o., not obtained; MGMT, O^6^-methylguanine-DNA-methyltransferase; TTFields, Tumor Treating Fields; Stupp, Chemotherapy following the Stupp procedure; CeTeG, Chemotherapy following the CeTeG procedure; TMZ, Temozolomide; PFS, Progression-free survival; CCNU, Lomustine; OS, overall survival.

|  | **Initial cohort (Essen)** | | **Verifiation cohort (Bonn)** | |
| --- | --- | --- | --- | --- |
|  | **n** | **%** | **n** | **%** |
| **Regrowth pattern^1^** |  |  |  |  |
| Local recurrence (after gross-total resection) | 63 | 46.3 | 31 | 27.4 |
| Local recurrence (progression of residual tumor) | 58 | 42.6 | 49 | 43.4 |
| Distant recurrence | 9 | 6.6 | 7 | 6.2 |
| New CE lesion in  prior non-CE lesion | 6 | 4.4 | 7  19 | 6.2  16.8 |
| Multicentric^2^ |  |  |  |  |
| **In case of local recurrence (after gross-total resection)** |  |  |  |  |
| Unifocal lesion | 55 | 84.6 | 7 | 22.6 |
| > 1 focal lesion | 5 | 7.7 | 16 | 51.6 |
| Entire margin | 5 | 7.7 | 8 | 25.8 |
| **In case of distant recurrence** |  |  |  |  |
| Unifocal lesion | 7 | 63.6 | 4 | 57.1 |
| > 1 focal lesion | 4 | 36.3 | 3 | 42.9 |

**Table S2.** Relapse growth patterns of GBM patients from the initial and verification cohort. ^1^Two patients with simultaneous local and distant regrowth were excluded from the initial cohort (Essen). ^2^ Simultaneous local and distant regrowth as an independent group excluded from other subgroups.

| **Variables** | **HR** | **95% CI** | **p-value** |
| --- | --- | --- | --- |
| MGMT promoter methylation | 0.51 | 0.31 – 0.83 | <0.01 |
| Chemo (Stupp) | 0.44 | 0.24 – 0.78 | <0.01 |
| Chemo (CeTeG) | 0.35 | 0.17 – 0.71 | <0.01 |
| Unifocal local recurrence (after gross-total resection) | 0.48 | 0.33 – 0.71 | <0.01 |
| Multifocal local recurrence (after gross-total resection) | 0.19 | 0.07 – 0.51 | <0.01 |
| Multicentric growth | 4.85 | 1.06 – 22.22 | 0.04 |

**Table S3.** Multivariate analysis of independent predictors/associations with PFS of our initial cohort (Essen). Cox regression model, stepwise backward elimination. Abbreviations: PFS, progression-free survival; HR, hazard ratio; CI, confidence interval.

| **Variables^1^** | **HR** | **95% CI** | **p-value** |
| --- | --- | --- | --- |
| MGMT promoter methylation | 0.36 | 0.24 – 0.55 | <0.01 |
| Radiotherapy (6 weeks) | 0.40 | 0.25 – 0.63 | <0.01 |
| Local recurrence (after gross-total resection) (yes) | 0.49 | 0.34 – 0.72 | <0.01 |
| Surgical intervention after recurrence (yes) | 0.55 | 0.38 – 0.80 | <0.01 |
| Radiotherapy after first recurrence (yes) | 0.60 | 0.38 – 0.94 | 0.03 |

**Table S4.** Multivariate analysis of independent predictors for OS of our initial cohort (Essen). Cox regression model, stepwise backward elimination. Variables included are stated in the Materials and Methods section. **^1^**Age (> 65 years) was eliminated in the last step of the model (p=0.052). Abbreviations: OS, overall survival; progression-free survival; HR, hazard ratio; CI, confidence interval.

| **Variables^1^** | **HR** | **95% CI** | **p-value** |
| --- | --- | --- | --- |
| MGMT promoter methylation | 0.31 | 0.19 – 0.13 | <0.01 |

Table S5. Multivariate analysis of independent predictors/associations with PFS of our verification cohort (Bonn). Cox regression model, stepwise backward elimination. Variables included are stated in the Materials and Methods section. ^1^Unifocal local recurrence (after gross-total resection) (yes) was eliminated in the last step of the model (p=0.062).
Abbreviations: PFS, progression-free survival; HR, hazard ratio; CI, confidence interval.

| **Variables** | **HR** | **95% CI** | **p-value** |
| --- | --- | --- | --- |
| Sex (female) | 0.58 | 0.34 – 0.99 | 0.04 |
| MGMT promoter methylation (yes) | 0.35 | 0.18– 0.68 | <0.01 |
| Unifocal local recurrence (after gross-total resection) (yes) | 0.46 | 0.22 – 0.96 | 0.04 |
| Multicentric recurrence (yes) | 2.76 | 2.45 – 5.25 | <0.01 |
| Surgical intervention after recurrence (yes) | 0.34 | 0.14 – 0.81 | 0.02 |

**Table S6.** Multivariate analysis of independent predictors for OS of our verification cohort (Bonn). Cox regression model, stepwise backward elimination. Variables included are stated in the Materials and Methods section.
Abbreviations: OS, overall survival; progression-free survival; HR, hazard ratio; CI, confidence interval.
